# Cholera outbreak in 2022 among children in Karachi: Study of cases attending to a Tertiary Care Hospital

**DOI:** 10.12669/pjms.39.5.7395

**Published:** 2023

**Authors:** Samina Junejo, Saba Shahid, Nazia Khursheed, Sidra Maqsood, Fareeha Adnan, Fatima Khalid, Qurat-ul-Ain Zahid

**Affiliations:** 1 Samina Junejo, Department of Pediatrics, Indus Hospital & Health Network, Karachi, Pakistan; 2 Saba Shahid, Department of Pediatrics, Indus Hospital & Health Network, Karachi, Pakistan; 3 Nazia Khursheed, Department of Microbiology, Indus Hospital & Health Network, Karachi, Pakistan; 4 Sidra Maqsood, Research Department, Indus Hospital & Health Network, Karachi, Pakistan; 5 Fareeha Adnan, Department of Microbiology, Indus Hospital & Health Network, Karachi, Pakistan; 6 Fatima Khalid, Department of Pediatrics, Indus Hospital & Health Network, Karachi, Pakistan; 7 Qurat-ul-Ain Zahid, Department of Microbiology, Indus Hospital & Health Network, Karachi, Pakistan

**Keywords:** Cholera, *V. cholerae*, Children, Diarrhea, O1 Ogawa, Inaba. Recent outbreak

## Abstract

**Objective & Background:**

Repeated outbreaks of cholera have occurred in Karachi. Changing patterns in seasonality, serotypes and antibiotic resistance have been observed in these outbreaks. Recently, in the year 2022, a surge of cholera cases has been reported from Karachi during the months of April-June. This study aimed to identify clinical features, antibiotic susceptibility, complications, and response to treatment of *V. cholerae* infection among children attending Indus hospital, Karachi.

**Methods:**

A retrospective chart review of pediatric patients was conducted for children aged 0-16 years. All children treated for culture-proven cholera infection at Indus Hospital from March to June 2022 were included. Details of clinical features, complications, antibiotic susceptibility, and response to treatment were retrieved from the health management information system (HMIS) of the hospital.

**Results:**

Twenty children were included. The median age was 01 (0.50-3.75) years. There were 9 (45%) males and 11 (55%) females. All the culture isolates belonged to serogroup O1 Ogawa of the *Vibrio cholerae*. Vomiting and diarrhea were the most common symptoms. Dehydration, acute kidney injury, and shock were seen in 19 (95%), 6 (30%), and 2 (10%) children respectively. Eleven children were admitted with an average hospital stay of 5 (Median-IQR 3-6) days. The isolates were completely susceptible to tetracycline, ciprofloxacin, and azithromycin. Different antibiotics were given which included cefotaxime, ceftriaxone, doxycycline, and ciprofloxacin. All children responded completely to the antibiotics.

**Conclusion:**

In present study all *V*. *cholerae isolates belonged to* the O1 Ogawa serotype that showed complete susceptibility to tetracycline, ciprofloxacin, and azithromycin. Dehydration, electrolyte imbalance, and renal impairment were the most common complications observed. Drinking unboiled water was identified as a potential source of cholera in most children. Therefore, advocacy of hygienic practices and disinfection of water supplies is recommended to prevent future cholera outbreaks

## INTRODUCTION

Cholera is an acute diarrhea illness caused by the ingestion of the bacterium *Vibrio cholerae*.^1^ This gram-negative bacterium has many serotypes, based on its O-antigen. Serotypes O1 and O139 are usually associated with cholera epidemics. The O1 serogroup is further subclassified into El Tor and Classical biotypes.^2^ Over the years, repeated outbreaks of *V. cholerae* have been recorded in Pakistan. A major number of cases are reported from Karachi. According to a recent report, there are more than 600 slums in Karachi accommodating millions of people.^3^ Such high number of slums lead to overcrowding, poverty, the unplanned urban infrastructure of the city, and peri-urbanization.^4^,^5^ which lead to cholera infections. Another major reason for cholera outbreaks in Karachi is the inferior quality of drinking water. According to a report, 91% of Karachi’s water supply is contaminated with Shigella, *E. c*oli, and *V. cholerae*.^6^ Furthermore, due to increasing poverty, the purchase of safe water, boiling and filtration of drinking water is not a sustainable alternative for most people. As a result, many people utilize tap or underground water for drinking purposes. These prevailing conditions have resulted in many cholera outbreaks in Karachi which are defined by Global task force as an unexpected increase in number of suspected cholera cases, over 2 consecutive weeks, of which some are laboratory confirmed.^7^

An epidemiologic review of cholera outbreaks in Pakistan shows that an epidemic of *V. cholerae* O1 Ogawa occurred in 1993 followed by an outbreak of O 139 serotype in 1994.^8^ Since then, outbreaks have been reported periodically in the years 2001, 2005, 2010, and 2015.^4^,^9^,^10^ Furthermore, literature from Pakistan shows that cholera outbreaks have been observed both in the rainy and dry seasons with sporadic distribution involving different range of geographic locations.^4^ A review of cholera serotypes shows that O1 serogroup is the predominant *V. cholerae* infection in Pakistan, while O 139 serotype infection occurs sporadically and generally involve older populations.^11^ Recently Ogawa and Inaba are emerging as most frequent serotypes.^9^

In the year 2022, a surge of cholera cases has been reported from Karachi during the months of April-June. Sindh health department confirmed 129 cases of cholera so far from the South, East, and Central districts of Karachi.^3^ Indus Hospital, Korangi is located in the East district of Karachi, we treated a considerable number of diarrhea cases in the pediatric department of the hospital since April 2022. Our lab confirmed many cases of cholera both in adults and children. Therefore, this study is undertaken to identify clinical features, antibiotic susceptibility, complications, and response to treatment of *V. cholerae* infection among children attending Indus Hospital. We also aim to compare antimicrobial susceptibility pattern of *V.cholerae* in the present study with the previously reported antibiotic susceptibility pattern.

## METHODS

This study is a retrospective chart review. All culture-confirmed cases of cholera reported from the pediatric department of the Indus Hospital from March to June 2022, who had at least one follow up after treatment, were included in the analysis. Records of children with ages ranging from 0-16 years were included. Suspected cases or confirmed cholera cases who did not come for follow up were excluded. Cases were enrolled from emergency and outpatient departments as well as from the ward. The data was collected when there was cholera outbreak, during this time it was hospital policy to send stool culture and serogroup analysis in all children who came with acute gastroenteritis. Stool microscopy and culture reports collected at the time of initial visit was included in the analysis. Details of clinical features, complications, antibiotic susceptibility, and response to treatment were retrieved from the HMIS of the hospital. Severe dehydration was labeled when signs like sunken eyes, delayed skin turgor or delayed capillary refill were present. Dehydration was classified according to integrated management of childhood illness (IMCI)^12^ Mild or moderate dehydration was considered when there was irritability, increased thirst, or electrolyte imbalances. Acute kidney injury (AKI) in our study was identified by using the creatinine clearance criteria (eCCl) of pRIFLE classification.^13^ Estimated creatinine clearance (eCCl) was calculated using the Schwartz formula.^14^ The children were assumed to have normal renal function and were assigned a baseline eCCl range of 75-120 mL/min/1.73 m^2^.^15^ Reduction of eCCl by 25% was considered a risk of renal injury while eCCl reduction of 50% was considered renal injury.^13^

### Ethical Approval:

This study bearing approval number IHHN_IRB_2022_07_001, dated 1^st^ July 2022, was approved by the hospital before starting the study. The standard treatment for all cases included fluid and electrolyte replacement. Antibiotics were used in most cases.

### Laboratory Techniques:

Stool samples of patients presenting with watery diarrhea were included. Samples were processed according to the standard operating procedure by following the American Society for Microbiology (ASM) guidelines. Samples were inoculated on Thiosulfate-citrate-bile-salt-sucrose (TCBS), Xylose-lysine deoxycholate (XLD), MacConkey agar (MAC) agar, and alkaline peptone water (APW). Isolates were sub cultured after overnight incubation to TCBS to enhance the recovery of pathogen. After Incubation at 35±2ºC, ambient air for 24-48 hours, suspected colonies from TCBS were gram stained and streaked on nutrient agar and incubated at 35±2ºC ambient air for 24 hours. Biochemical tests were performed and API 20 E strip (Biomérieux, Lyon, France) was used for definitive diagnosis. Serotyping was performed using polyvalent anti-serum to identify O1and O139 strains and further into Ogawa and Inaba serotypes (Dienka Sieken Co. Limited Japan). Antimicrobial susceptibility testing was performed on Mueller Hinton agar (MHA) using the Kirby-Bauer disk method. The panel of antibiotics tested includes ampicillin (AMP), tetracycline (TE), ciprofloxacin (CIP), trimethoprim/sulfamethoxazole (SXT), doxycycline (DO), and azithromycin (AZM). Latest CLSI guidelines were used to interpret susceptibility results except for AZM which was measured according to the guidelines of the European Committee on Antimicrobial Susceptibility Testing^16^

### Statistical Analysis:

Data were analyzed using the Statistical Package for Social Studies (SPSS; IBM, version 24.0 Corporate headquarters one New Orchard Road Armonk, New York 10504-1722 United States). Median (Interquartile range IQR) was computed for age, days of hospital stay, serum electrolytes, creatinine, hemoglobin, total leukocyte count (TLC), neutrophils, lymphocyte, and platelets, as data were not normally distributed. Frequencies with percentages were computed for sex, district, source of water, frequency of stool per day,response to treatment and symptoms.

## RESULTS

A total of 20 cases were included. The median (IQR) age of children was 01 (0.50-3.75) years. There were 9 (45%) males and 11 (55%) females (Table-I). Majority of cases 12 (60%) were from Korangi followed by Karachi East 6 (30%) (Table-I). Thirteen (65%) children had history of consumption of un-boiled tap water. Among the clinical variables reviewed, most of the children, 12 (60%) had diarrhea with 8-9 episodes of stools per day. Renal function was assessed in ten children, out of which six had renal involvement. Five out six children had acute kidney injury (AKI) with decreased urine output and 50% reduction of creatinine clearance. Children with AKI had an average hospital stay of 4 days compared to hospital stay of 2.8 days in children who did not have any renal involvement. In follow up visit, renal function of all the children recovered completely (Table-I).

**Table-I:** Demographic and clinical characteristics of the study participants.

Demographic details	

Age	Median (IQR)
Median (IQR) (year)	01 (0.50-3.75)
***Gender (n) (%)***	n (%)
Male	09 (45)
Female	11 (55)
***District (n) (%)***	***n (%)***
Karachi Central	01 (05)
Karachi East	06 (30)
Karachi West	01 (05)
Korangi	12 (60)
***Source of water consumption (n) (%)***	***n (%)***
Unboiled water bought from outside	07 (35)
Unboiled tap water	13 (65)
Boiled water used	5 (25)
Filtered water used	6 (30)
***Clinical Details (n) (%)***	
***Stool frequency per day***	
3-4	01 (05)
5-6	02 (10)
7-13	05 (25)
Multiple>13	12 (60)
***Symptoms***	
Fever	06 (30)
Vomiting	16 (80)
Blood in stool	02 (10)
Shock	02 (10)
Fits	01 (05)
Dehydration	19 (95)
Mild	04 (22)
Moderate	09 (47)
Severe	06 (31)
Renal involvement (6)	
Risk of renal injury	1(10)
Renal injury	5 (50)
Hospital stay in days	Median (IQR)

***Median (IQR)***	***05 (03-06)***

***Laboratory details Median (IQR)***	
Serum Potassium	3.0 (2.55-3.55)
Creatinine	0.82 (0.48-1.33)
Creatinine clearance	49.83 (28.67-70.53)
Chloride	106 (100-108)
Bicarbonates	9.95 (8.25-13.4)
Hemoglobin	10.70 (8.45-12.85)
Total Leucocyte count	12.80 (9.15-17.60)
Neutrophils %	61.25 (58.55-73.45)
Lymphocyte %	27.30 (20.77-36.32)
Platelets	470.5 (365-629.75)
Stool findings n (%)	n (%)
Multiple pus cells present	8 (40)
Presence of RBC	2 (10)
Outcomes n (%)	n (%)
Admitted	11 (55)
Managed in outpatient department	6 (30)
Referred out	3 (15)
Cured	20 (100)

Sixteen (80%) children presented with vomiting followed by fever noted in 6 (30%) children. Two (10%) children had dysentery. Shock and fits were seen in 2 (10%) and 1 (5%) cases of the children, respectively. Dehydration was seen in 19 (95%) patients, out of which, 6 children (31%) suffered from severe dehydration. Rehydration was done by using normal saline (N/S) or ringer lactate (R/L) or combination of the two fluids (Table-II).

**Table-II:** Complications and details of treatment given to study participants.

Medical condition n (%)	Treatment given
Severe dehydration 6 (31)	All required I/V boluses+ maintenance fluid
Moderate dehydration 9 (47)	Maintenance fluid with Saline or Ringo-lactate+ ORS
Mild dehydration 4 (22)	ORS
Hypokalemia 4 (20)	Intravenous potassium replacement (4 children)
Acidosis 15 (75)	Bicarbonate (5 children) + rehydration
Excessive vomiting 6 (30)	Antiemetics
Details of antibiotics 16 (80)	Doxycycline (4 children)
	Ciprofloxacin (6 children)
	Ceftriaxone (4 children)
	Cefotaxime (2 children)

Acidosis was identified in 15 (75%) children out of which five children required bicarbonate replacement in addition to hydration therapy. A total of 13 (65%) children got admitted with a median (IQR) hospital stay of five (3-6) days (Table-II). Cefotaxime and ceftriaxone alone were given to four children. Two children received combination of ceftriaxone and ciprofloxacin while ten children were given either ciprofloxacin or doxycycline alone (Table-II). All children responded to the antibiotics, however children who were given antibiotics on basis of antimicrobial susceptibility pattern (ciprofloxacin or doxycycline) had shorter hospital stay of 2-3 days compared to children who received empirical treatment (cefotaxime or ceftriaxone) who had hospital stay of 4-5 days.

The serological analysis revealed that all the isolates belonged to serogroup O1 Ogawa of *V. cholerae*. Analysis of month-wise distribution showed that 20% of cases were reported in March while 80% of cases were in the month of April and May, (Fig.2).

**Fig.1:**
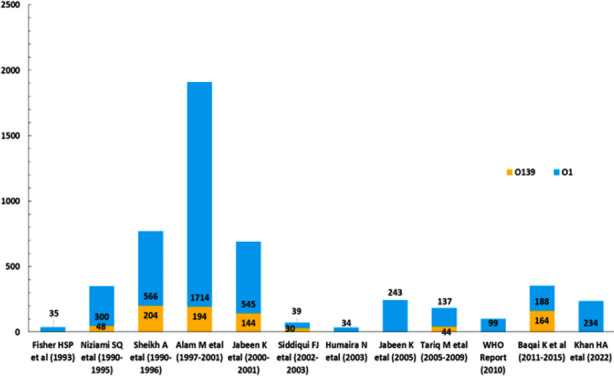
Outbreak frequency of V. cholerae strains O1 and O139 from 1993 to 2022 in Karachi.

**Fig.2:**
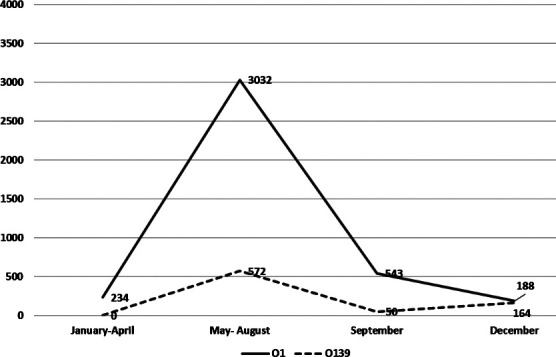
Month-wise distribution of *V. cholerae* strains O1 and O139 from 1995 to 2022 in Karachi.

The bacterial *V. cholerae* isolates were assessed for antimicrobial susceptibility pattern against the commonly used antibiotics. Maximum resistance was seen against trimethoprim/sulfamethoxazole (75%) followed by ampicillin (40%). We observed 100% susceptibility to azithromycin, doxycycline, ciprofloxacin, and tetracycline. A comparison of antibiotic susceptibility between the present and past studies shows continued resistance of *V.cholerae* to co-trimoxazole and ampicillin, while the resistance to tetracycline has declined. Resistance to doxycycline was not reported in any past local study, however, we found no resistance to doxycycline in the present study. The antibiotic susceptibility pattern is given in Table-III.

**Table-III:** Antimicrobial resistance patterns against V. cholerae from present study and comparison to other studies.

Antibiotics	Fisher HSP et al (1993)	Sheikh A et al (1990-1996)	Jabeen K et al (2000-2001)	Baqai K et al (2011-2015)	Present study
Chloramphenicol	-	21.83%	0%	2.6%	-
Co-trimoxazole	100%	94.9%	0%	66.2%	75%
Tetracycline	0%	29.3%	-	40.9%	0%
Doxycycline	-	-	-	0%	0%
Ampicillin	-	-	-	8.8%	40%
Ciprofloxacin	-	-	-	1.4%	0%
Erythromycin	-	67.8%	-	-	-
Azithromycin	-		-	-	0%
Nalidixic Acid	-	0.98%	-	-	-

## DISCUSSION

Serogroups and serotypes of *V. cholerae* vary widely in cholera pandemic, epidemic and outbreaks worldwide. There was much variation in frequency of *V*. cholerae O1 and O 139 cases during several outbreaks in Karachi from 1993 to 2022 (Fig.1). However, serotype O1 Ogawa remains more prevalent in Karachi^17^ (Fig.1). Another important epidemiological finding to be noted is switching of *V. cholerae* serotypes over the years. Previously prevalent *V. cholerae* O1 Ogawa was replaced by O1 Inaba serotype in 2005^17^ and in years 2011-2015.^10^ Our study however reported predominant O1 Ogawa again. One possible explanation for shift of serotypes from Inaba to Ogawa could be environmental contamination with predominantly Ogawa serotype, as was observed in Haiti.^18^ Another explanation for decreased Inaba infection could be acquisition of immunity in general population, as Inaba serotype is found to be more immunogenic and results in longer-term protection following infection.^19^

Since last two decades there is no specific seasonal pattern of cholera infections in Pakistan. Intervals of sporadic cases with localized outbreaks have been reported throughout the year (Fig.2). We encountered maximum number of cases in May-August, which coincides with the time in which O1 Ogawa infection was previously reported (Fig.2). During the study period average rainfall and temperature were unfavorable for cholera outbreak and seem less likely to be responsible for increased number of cases.

Wastewater environmental surveillance^20^ of toxigenic *V*. cholerae conducted by the National Institute of Health- akistan in 2019-2022 found high load of toxigenic cholera strains in Karachi from May 2019-February 2020. Although water surveillance was not done during the study period, considering poor sanitation conditions in Karachi, it is possible that environmental load of *V*. *c*holerae is still high which led to cholera outbreak during May till August 2022.

Epidemiological data shows that frequency of cholera outbreak is on the rise in Pakistan and particularly in Karachi. It is therefore essential to vigilantly follow up on cholera cases by means of an efficient way such as waste water surveillance. This will not only mitigate the need for large population testing but will also help in predicting cholera outbreaks.

In present study, five children consumed boiled water while six children had access to filtered water. Remaining children consumed water without any sterilization, indicating infected water could be possible source of infection (Table-I.)

Centre for Disease Control and Prevention (CDC) recommends boiling of water for one minute, chlorination of water or use of household bleach to remove *V. cholerae* contamination.^21^ along with other strategies like washing of hands and safe disposal of stools. Rigorous implementation of preventive strategies as recommended by CDC can prevent future outbreaks of cholera. We strongly advocate educating people about improving hygiene and disinfection practices of water supplies. Various forums like media, schools, and health care facilities can take the lead for advocacy of safe water use.

Although fluid resuscitation is the mainstay of treatment in cholera, however, use of antimicrobials can shorten duration of illness by diminishing production of the cholera toxin^22^ Furthermore, antibiotics can play an important role during cholera outbreaks by diminishing the volume and frequency of stools approximately by 50%.^23^

While prescribing antibiotics for cholera one important consideration is dynamic bacterial susceptibility of *V. cholerae*, which may increase or decrease depending on antibiotic consumption and the emergence of new serotypes. Some of the antibiotics previously noted to be resistant may become relevant again such as tetracycline.^24^ Evidence shows that antibiotic susceptibility of *V. cholerae* in Karachi has changed over a period of time.^4^,^9^,^10^ Table-III. Similarly, we observed change in antibiotic susceptibility pattern. No resistance to tetracycline, which was previously reported to be 41%. was noted. Furthermore, resistance to ciprofloxacin or azithromycin was nil (Table-III). Change in antibiotic susceptibility pattern could be due to presence of distinct phenotypes and genetic traits in serotype O1 and O139. Genes conferring resistance to antibiotics are transferred to *V.cholerae* via plasmids, gene cassettes and mobile genetic elements and create antibiotic resistance in environmental reservoir.^25^

WHO recommends 20 mg oral zinc sulphate per day for ten days for cholera infection along with antibiotics in severe cases.^24^ Doxycycline 2-4 mg/kg orally as a single dose is recommended, alternatively azithromycin or ciprofloxacin 20mg/kg orally as a single dose can be prescribed.^25^ In Pakistan doxycycline is not available in syrup form and needs to be formulated through pharmacy. Additionally, judicious use of azithromycin is advised in our setup due to widespread prevalence of extensively drug resistant (XDR)-enteric infection. Considering this fact, ciprofloxacin may prove to be a prudent choice of antibiotic for treating cholera in our context.

We encountered unusually high number of gastroenteritis cases in March, initially children were given cefotaxime or ceftriaxone empirically, however as cultures grew *V. cholerae*, antibiotics were given according to culture and sensitivity report. As bacterial isolates were completely sensitive to ciprofloxacin or doxycycline, so both the drugs were used in our institute. Admitted children were given a single dose of doxycycline while children managed on OPD basis received a single dose of ciprofloxacin. Six children received antibiotics other than ciprofloxacin and doxycycline, which included cefotaxime and ceftriaxone, although these antibiotics were not included in the susceptibility panel. However, cefotaxime or ceftriaxone were not changed as children recovered before the culture report came. Twelve children received either ciprofloxacin or doxycycline alone or in combination with ceftriaxone. Although all children in our study recovered completely but those children who received single antibiotic based on antimicrobial sensitivity had an average hospital stay of 2-3 days, compared to a hospital stay of 4-5 days in children who received ceftriaxone or cefotaxime. However, the difference in hospital stay could not be statistically proven due to small sample size. This observation in our study reinforces results of other studies^23^,^26^ that report reduce duration of illness and diarrhea with usage of effective antibiotics.

We observed AKI in six children. Similarly, renal injury in cholera infection has been reported in other studies.^26^ It is mostly related to intravascular volume contraction which leads to decrease glomerular filtration of kidneys.^25^ WHO recommends ringers lactate as first choice of fluid because it significantly decreases acidosis and renal impairment.^26^ We strongly advocate using ringers lactate as standard rehydration fluid in cholera infection as there is considerable risk of acidosis and renal injury during acute illness.

### Limitations:

This study included a small sample size from a tertiary care hospital, which was not representative of the general population. Well-designed studies with larger sample size are needed to assess clinical features, complications, drug resistance and treatment response trend of cholera infection in general population.

## CONCLUSION

In present study, all the *V*. *cholerae isolates belonged to* the O1 Ogawa serotype and showed complete susceptibility to tetracycline, ciprofloxacin, and azithromycin. Dehydration, electrolyte imbalance, and renal impairment were the most common complications observed. Majority of the cases had history of consumption of unboiled water, indicating infected water may be possible source of infection. Advocacy of hygienic practices and disinfection of water supplies is recommended to prevent future cholera outbreaks.

### Authors’ contributions:

**SJ, SS and NK:** Conceived idea of the study and participated in study design and write up.

**FA, FK and QAZ:** Carried out data collection, SM assisted with statistical analysis.

All authors were involved in the coordination of the study, drafting the manuscript and approving the final version.
